# Introduction of a New Surgical Method to Improve Bone Healing in a Large Bone Defect by Replacement of the Induced Membrane by a Human Decellularized Dermis Repopulated with Bone Marrow Mononuclear Cells in Rat

**DOI:** 10.3390/ma13112629

**Published:** 2020-06-09

**Authors:** Maximilian Leiblein, Tobias Kolb, Lion Christian, Katrin Schröder, Ceyhan Yaman, Alexander Schaible, Ingo Marzi, Dirk Henrich, Maren Janko

**Affiliations:** 1 Department of Trauma, Hand and Reconstructive Surgery, University Hospital, Goethe University Frankfurt am Main, 60590 Frankfurt, Germany; tobias_kolb@gmx.de (T.K.); lion.christian@online.de (L.C.); office@trauma.uni-frankfurt.de (C.Y.); alexanderschaiblex@gmail.com (A.S.); marzi@trauma.uni-frankfurt.de (I.M.); D.henrich@trauma.uni-frankfurt.de (D.H.); maren.janko@kgu.de (M.J.); 2 Center of Physiology, Cardiovascular Physiology, University Hospital, Goethe University Frankfurt am Main, 60590 Frankfurt, Germany; schroeder@vrc.uni-frankfurt.de

**Keywords:** bone healing, bone marrow mononuclear cells, human decellularized dermis, induced membrane

## Abstract

The Masquelet technique for the treatment of large bone defects is a two-stage procedure based on an induced membrane. We eliminate the first surgical step by using a decellularized dermal skin graft (Epiflex^®^) populated with bone marrow mononuclear cells (BMC), as a replacement for the induced membrane. The aim of this study was to demonstrate the feasibility of this technology and provide evidence of equivalent bone healing in comparison to the induced membrane-technique. Therefore, 112 male Sprague–Dawley rats were allocated in six groups and received a 10 mm femoral defect. Defects were treated with either the induced membrane or decellularized dermis, with or without the addition of BMC. Defects were then filled with a scaffold (β-TCP), with or without BMC. After a healing time of eight weeks, femurs were taken for histological, radiological and biomechanical analysis. Defects treated with Epiflex^®^ showed increased mineralization and bone formation predominantly in the transplanted dermis surrounding the defect. No significant decrease of biomechanical properties was found. Vascularization of the defect could be enhanced by addition of BMC. Considering the dramatic reduction of a patient’s burden by the reduced surgical stress and shortened time of treatment, this technique could have a great impact on clinical practice.

## 1. Introduction

Critical size bone defects are defined by a loss of more than two centimeters or more than 50% of the bone’s circumference [1,2] and are still a major challenge in orthopedic and trauma patients. As gold standard for their treatment, autologous bone grafts taken from the iliac crest and fibula are considered. However, in patients with comminuted fractures, nonunion, peri-prosthetic fractures, infection or tumor resection, the resulting defects are large and the volume of bone available for grafting is limited. Further limitations associated with bone grafts taken from the iliac crest include fractures during harvesting and postoperative donor site pain [3]. As an alternative to current methods of filling large bone defects, Masquelet developed a two-step procedure based on an induced membrane encapsulating the defect. In the first step, a polymethylmethacrylate (PMMA) spacer is inserted into the defect, which, over a period of 8 to 12 weeks, causes a membrane to form. In the second step, the membrane is opened, the PMMA spacer is removed and the defect is filled with autologous bone. Utilizing this technique, Masquelet and others have described complete bone healing and restoration of limb function [4,5].

The synovial-like induced membrane that forms around the cement spacer is described as being 1–2 mm thick, highly vascularized, and protective against bone graft resorption, with conditions favoring bone healing. In previous work, our group reproduced Masquelet’s technique in a 10 mm plate-stabilized femoral bone defect in rat and analyzed the kinetics of the induced membrane formation.

In spite of the positive clinical results achieved using the induced membrane technique, it still requires large quantities of autograft bone. We previously elucidated the influence of different regenerative cells, such as marrow stromal cells (MSC) and cell mixtures such as bone marrow mononuclear cells (BMC) transplanted into the membrane envelope in comparison to syngeneic bone on bone healing, using the same induced membrane rat model [6]. Syngeneic bone had the greatest impact on bone healing; however, defects treated with regenerative cells were oftentimes comparable [7]. This effect is presumably caused by the regenerative potential of monocytes and hematopoietic cells within the BMC preparation, leading to an increased neovascularization and providing osteogenic factors [8,9,10,11,12].

Epiflex^®^ is a decellularized human dermal graft, suitable as a matrix for tissue regeneration and cell infiltration [13]. It is approved for clinical application, with indications such as chronic wounds, burns [14,15], hernia surgery [16,17] or breast reconstruction. Furthermore, it was shown that wound healing can be improved by populating the dermis with regenerative cells [18].

The induced membrane technique requires multiple surgeries, which increases patients’ burden and health costs. A solution to this might be to omit the first surgical intervention by replacement of the induced membrane by a tissue-engineered construct. Here, we report about the effect of a decellularized commercial dermal graft repopulated with bone marrow mononuclear cells, as replacement of the induced membrane in comparison to the induced membrane technique on the healing of a large (10 mm) femoral bone defect of rat, filled with β-TCP with or without BMC.

## 2. Materials and Methods

### 2.1. Animal Care and Experimental Groups

All animal experiments were carried out in compliance with regulations stated by The Institutional Animal Care and Oversight Committee at ‘Regierungspräsidium, Darmstadt, Germany’, following the ARRIVE guidelines (Project No. FK10/57), in accordance with German law.

One-hundred and twelve 8 to 10-week old male Sprague–Dawley (SD) rats (Harlan-Winkelmann, Horst, the Netherlands), weighing approximately 250–300 g were housed in individual cages, in temperature 21.8 °C, air flow and light-controlled (12 h day and 12 h night) rooms. The animals received rat food and water ad libitum. For 5 days, animals were postoperatively monitored daily for signs of pain, discomfort and complications.

### 2.2. Treatment Groups

Six groups were evaluated: (1) induced membrane filled with β-TCP (Chronos, 0.7–1.4 mm, DePuy Synthes, Umkirch, Germany); (2) induced membrane filled with β-TCP loaded with BMC; (3) decellularized dermis filled with β-TCP; (4) decellularized dermis filled with β-TCP loaded with BMC; (5) BMC-loaded decellularized dermis filled with β-TCP; (6) BMC-loaded decellularized dermis filled with β-TCP loaded with BMC. Each group consisted of 16 animals. Healing time was 8 weeks. Additionally, 16 animals were sacrificed and used as donors for syngeneic bone marrow-derived mononuclear cells (BMC) (Table 1).

### 2.3. Bone Marrow-Derived Mononuclear Cells (BMC)

Bone marrow was isolated from rat femora obtained from syngeneic donor rats (SD rats), as described previously [7,11,19]. In brief, femora were removed, condyles were cut and bone marrow was flushed with a sterile syringe filled with PBS supplemented with 1% penicillin and streptomycin (Biochrom, Berlin, Germany). The cells were recovered, washed once with PBS and layered on a *Ficoll* density gradient and centrifuged continuously at 800 g for 20 min. Mononuclear cells were collected, washed three times with PBS (10 min, 800 g), re-suspended in PBS and counted.

BMC were seeded on β-TCP scaffolds, as described [7,10,12,20]. Scaffold granules were placed in a dense monolayer in individual wells (area = 1 cm^2^) of a 48-wellplate (Nunc, Wiesbaden, Germany), using sterile forceps. A number of 0.5 × 10^6^ BMC in a volume of 175 μL PBS was carefully dripped onto each scaffold, followed by 10 min incubation at 37 °C. Medium containing non-adherent cells was subsequently removed and dripped once again onto the biomaterials, followed by incubation, as indicated above. This procedure was repeated once again. The achieved density of BMC is approximately 1.3 × 10^6^ cells per cm^3^ scaffold, which is in accordance with our previous work [10,21,22].

**Figure 1:**
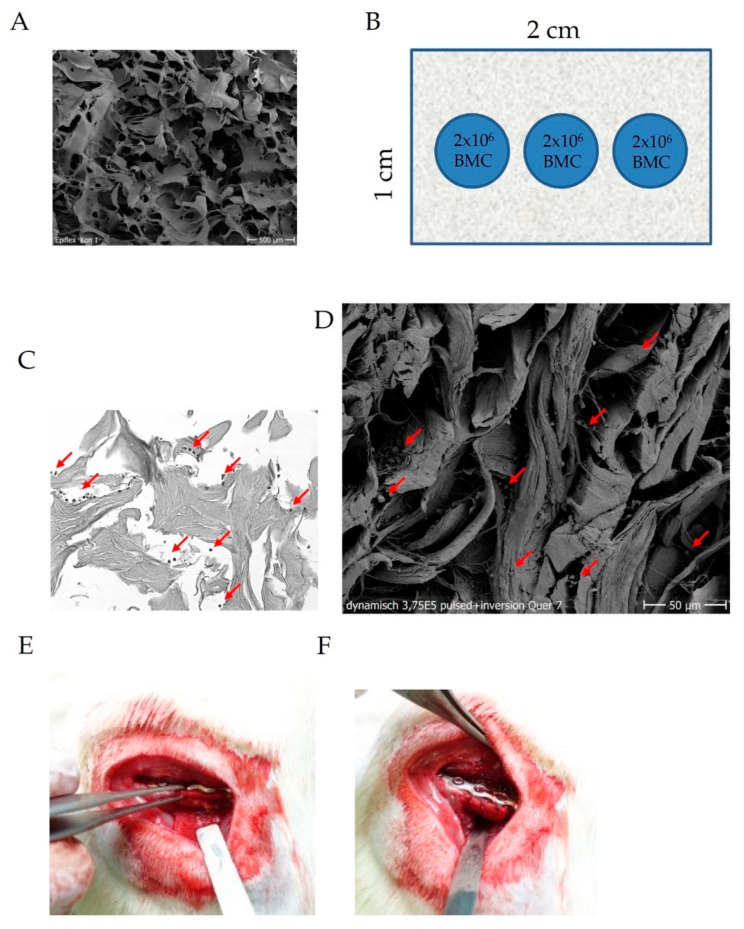
Internal structure of the decellularized dermis (**A**), cell seeding scheme (**B**), histological (HE-stain) and SEM proof of BMC (red arrows), within internal structures of the dermis (**C**,**D**) and fitting, filling and suturing of the replacement membrane (**E**,**F**).

Stripes of human decellularized dermal graft (1.2 cm × 1.8 cm) were placed upside down in a well plate and hydrated in PBS for 10min. Subsequently, BMC were seeded to the membrane applying a dynamic seeding procedure. In brief, BMC suspension (concentration: 2 × 10^6^/cm^3^ biomaterial) was dripped in three individual linearly arranged droplets (each 30 mL), covering the length of the membrane stripe followed by five centrifugation steps at 300× *g* for each 1 min (Figure 1B). Then, the membrane was inverted and BMC suspension was dripped on the membrane, as described, followed by centrifugation for 1 min at 300× *g*. The BMC loaded membrane was stored in PBS under sterile conditions at 37 °C until use (modified from [23]).

### 2.4. Surgical Procedures: Generation of the Induced Membrane, Implantation of Decellularized Dermal Graft and β-TCP Scaffolds

The femur critical size defect was induced as previously described [10,20,24,25]. In detail, rats were anesthetized by the intraperitoneal administration of Ketavet (70 mg/kg) and Rompun (10 mg/kg). Rat right hind limb was shaved, aseptically cleaned and animals were placed in a lateral position. A lateral longitudinal incision over the femur was made. The fascia was cut and the muscles were bluntly separated between the musculus quadriceps femoris and the hamstrings. A six-hole locking compression plate (Miniplate ‘Lockingplate LCP Compact Hand 1.5 straight’, DePuy Synthes, Dubendorf, Switzerland, catalog number 036.000.038) was placed into the anterior aspect of the femoral shaft. Four locking screws (1.5 mm) fixed the plate to the bone and the bone cortex was then cut by a Gigli saw (RISystems, Davos, Switzerland) and a bone critical size defect of 10 mm was created in the midshaft around the middle holes of the plate. In group 1 and 2, the defect was filled with a cylindrical PMMA spacer (Palacos R+G, Heraeus Medical GmbH, Wehrheim, Germany) for membrane induction. The cement was hand mixed according to the manufacturer’s protocol. The wound was irrigated with sterile saline, fascia re-approximated with interrupted 5-0 Vicryl sutures and the superficial fascia and skin closed with Prolene 5-0 suture (Ethicon, Germany). Animals were returned to their cages, monitored daily for the occurrence of abnormal behavior or complications, and analgesia was given for seven days postoperatively.

In a second surgical intervention, after three weeks the induced membrane surrounding the defect was incised, the PMMA spacer was softly removed and the bone ends were refreshed by gentle scraping. Subsequently, the defects were filled with the intended transplants. Afterwards, the membrane was closed with interrupted 5-0 Vicryl sutures and the wound was closed as previously described. The operative approach and postoperative aftercare were performed as in the initial operation.

In groups 3–6, the implantation of the decellularized dermis was performed directly after creating the bone defect. The dermis was placed underneath the stainless steel-plate and fixed by the tightening of the inner screws. Subsequently the dermal graft was wrapped around the defect, filled with the intended transplant, closed and fixed to the plate by a suture.

The animals were painlessly killed by an overdose of pentobarbital (500 mg/kg intraperitoneally) after eight weeks and weighed afterwards. Femora were then carefully dissected. Blinded with respect to treatment, all bones were examined macro- and microscopically for signs of infection, tumors and correct fixation of the plate. Findings were recorded and only samples without findings were further processed. Bone samples were wrapped in prewetted gaze and stored at −80 °C until further processing.

For µCT-analysis, femora were defrosted and stored in 70% alcohol. Subsequently, those bones were used for biomechanical testing. Contralateral bones were treated equivalently and served as reference for biomechanical evaluation. For histological analysis, femora were decalcified and fixed in 10% formalin solution for subsequent paraffin embedding.

### 2.5. Evaluation

#### 2.5.1. µCT Analysis

To assess callus volume and mineralization of the decellularized dermis, µCT analysis was performed on six femur bones per group, using a high-resolution in-vivo-micro-CT Skyscan 1176 (Bruker AXS, Karlsruhe, Germany). The long axis of the femur was lined up orthogonally to the axis of the X-ray beam (Al 0.5 mm; voltage: 50 kV; current: 500 µA; frame average: 7; rotation ra.: 180; rotation st.: 0.5). Isotropoic voxel size was 18 µm^3^. The region of interest was set on the bone defect. Two-dimensional CT-images were scanned and reconstructed using a standard convolution-back-projection procedure and stored in 3D arrays. An analysis of new bone formation was performed histomorphometrically on the basis of 2D reconstructed sagittal µCT images of the bone defects using the software Image J (Rasband, W.S., Image J, U.S. National Institutes of Health, Bethesda, MD, USA, https://imagej.nih.gov/ij/index.html).

#### 2.5.2. Biomechanical Testing

Bones that had already been used for µCT-evaluation were analyzed biomechanically, as previously described, in a destructive three-point bending method, using a material testing machine (Zwickiline Z5.0, Zwick-Roell, Ulm, Germany). Between both procedures, bones were stored in 70% ethanol. “Bend to failure” was performed by lowering a rod onto the femur, using a constant deflection rate of 0.1 mm/s. Load and deflection were recorded continuously. The distance between the settings was 20 mm and point of failure was defined as maximum load followed by a quick load reduction of 50%. Stiffness (slope of the elastic deformation part of the load/deformation curve) was then calculated using the software TestExpert II (Zwick-Roell). Measurements were normalized to the corresponding contralateral femora [10,11,26].

#### 2.5.3. Histological Analysis

For histological evaluation of bone maturation, bones were carefully defrosted and fixed in 10% Zinc-Formal-Fixx (Thermo Electron, Pittsburgh, PA, USA) over 20 h, followed by decalcification for 14 days in 0.25 M Trizma base (Sigma-Aldrich, Taufkirchen, Germany) and 10% EDTA (Sigma-Aldrich), pH-value 7.4. Decalcified bones were paraffin embedded and cut into sections (3 µm), parallel to their long axis. Movat’s pentachrome staining was performed as published by Garvey et al. [27], using a staining kit, according to the manufacturer’s instructions (Morphisto, Frankfurt, Germany).

Callus maturation was measured using immunohistochemistry via detection of osteocalcin, and vascularization via staining of α-smooth muscle actin (α-SMA). The sections were incubated with monoclonal mouse anti-rat osteocalcin (1 h at room temperature, final concentration 10 µg/mL, clone 1A4, antibody-ID: AB_300332, Abcam, Cambridge, UK), or monoclonal mouse anti-rat α-SMA (1 h at room temperature, final concentration 2 µg/mL, clone 1A4, antibody-ID: AB_262054, Abcam, Cambridge, UK).

As secondary antibody, a polyclonal HRP conjugated anti-mouse IgG (Simple Stain Rat MAX PO, Nichirei, Tokyo, Japan) was applied for 30 min, followed by incubation with 3-amino-9-ethylcarbazole (AEC, Sigma-Aldrich). Finally, a counterstain with hematoxylin was performed. An independent observer blinded to the group setup analyzed the samples. All slides were analyzed using light microscopy (Axioobserver Z1, Zeiss, Gottingen, Germany; Biorevo BZ-9000, Keyence, Neu-Isenburg, Germany), in combination with a computer-supported imaging picture analysis system (Axiovision, Zeiss). High resolution images depicting the whole defect zone in each case were created by automated stitching of multiple single frames covering the whole defect, using the software BZII Analyzer (Keyence). New bone formation, osteocalcin or α-SMA-positive area was then analyzed in the defect site using the software Image J, as described previously [19,28]. In order to prevent the misinterpretation of α-SMA-positive cells as blood vessels, α-SMA-positive events below 10 µm in diameter were excluded from the automated evaluation. Since the intensity of osteocalcin staining in the various tissues/biomaterials does not allow a fully automated image analysis, the area of osteocalcin positive new bone tissue was marked by an independent observer using the polygon tool of Image J and osteocalcin positive area was calculated in relation to the callus tissue in the defect zone accordingly [26].

### 2.6. Statistics

Results are presented as box plots of the median in diagrams or as mean and standard deviation in the description of the results. A nonparametric Kruskal–Wallis test with Bonferoni–Holm corrected Conover–Iman post-hoc analysis was used for comparisons between the groups, using the statistical software Bias 11.02 (Epsilon Verlag, Darmstadt, Germany). The power analysis was calculated in order to detect differences of bone formation in histological analysis (based on Movat’s pentachrome staining), between six experimental groups. Based on earlier comparable studies [7,10,26], a pooled standard deviation of 25% and a power of 80% were deemed to detect true differences in means of 45% between the treatment groups at a level of two-sided significance of 5%. For these reasons, a group size of n = 10 for histology groups was chosen. *p*-values less than 0.05 indicate statistically significant differences. *p*-values between 0.05 and 0.1 were rated as a statistical trend.

## 3. Results

### 3.1. Animal Care/Complications

A total number of 11 animals were excluded due to pin loosening (n = 8), or infection (n = 1); two animals died due to breathing arrest during surgery. The numbers of excluded animals per group were one in group 1, five in group 2, four in group 4, and one in group 6. No macroscopically visible side effects of the human acellular dermis were recorded.

### 3.2. Low Biomechanical Properties in All Treatment Groups

**Figure 2:**
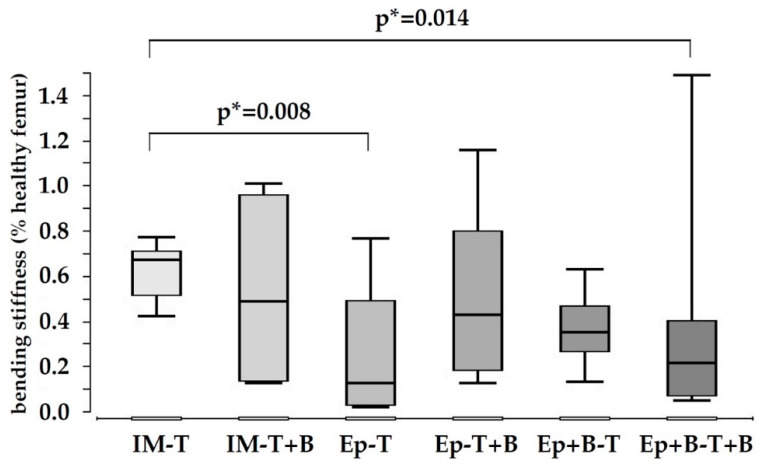
Bending stiffness of the 10 mm bone defect, in relation to the contralateral healthy femur (= 100%). No significant differences were detectable between the treatment groups. IM-T = induced membrane + β-TCP; IM-T + B = induced membrane + β-TCP loaded with bone marrow-derived mononuclear cells; Ep-T = Epiflex^®^ + β-TCP; Ep-T + B = Epiflex^®^ + β -TCP with BMC; Ep + B-T = Epiflex^®^ with BMC + β-TCP; Ep + B-T + B = Epiflex^®^ with BMC + β-TCP with BMC. p* = uncorrected *p*-value, indicates a statistical trend.

The biomechanical stability of the bone defect was analyzed in relation to the healthy contra-lateral femur. Bending stiffness was significantly lower in all treatment groups compared to control. Highest bending stiffness was measured in group 1 (induced membrane + β-TCP); however, the level of significance was not reached (Figure 2). Bending stiffness could not be increased by the addition of BMC.

Evaluating ultimate load by a destructive three-point bending test, treated bones showed low biomechanical properties and were frequently only bent, not fractured. As expected, contralateral healthy bones were fractured. Therefore, bending stiffness is presented, instead of ultimate load results.

### 3.3. Increased Bone Formation in Epiflex^®^-Groups with BMC

**Figure 3:**
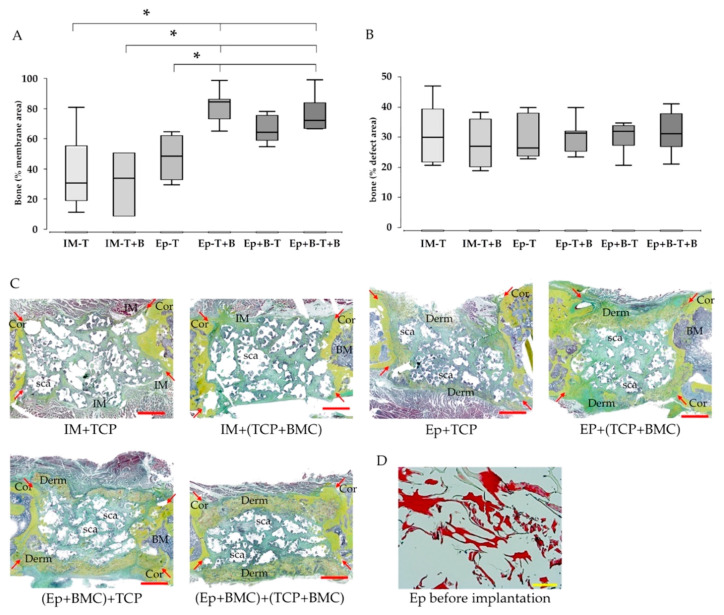
Evaluation of bone formation in the area of the induced membrane and decellularized dermis (**A**) and within the whole defect (**B**), based on Movat’s pentachrome stained histological slices. Significant increase of osseous transformation of the decellularized dermis was seen in groups treated with the decellularized dermis and additional presence of BMC. Representative images of Movat‘s pentachrome staining are shown in (**C**). Bone tissue appears yellow. As comparison, (**D**) shows the result of Movat‘s pentachrome staining of a native Epiflex^®^ before implantation. The dermis appears red. * = *p* < 0.05. Histology: Red bar represents 2 mm, yellow bar represents 100 µm, arrows represent initial fracture borders, BM = bone marrow, Cor = corticalis, Derm = decellularized dermis, IM = induced membrane, sca = scaffold.

An analysis of Movat’s pentachrome-stained histological slides revealed a significant increase of osseous transformation of the decellularized dermis in animals treated with Epiflex^®^ with BMC, compared to groups treated with Epiflex^®^ without BMC and induced membrane groups (Figure 3A). Untreated native Epiflex^®^ appears red in Movat’s pentachrome staining (Figure 3D), in situ Epiflex^®^ appears to be yellow, which is comparable to bone tissue that can particularly be found in the presence of BMC. Partially, the implanted dermis showed an osseous connection to the fracture site. In comparison to the induced membrane, Epiflex^®^ is distinctively thicker (Figure 3C). Callus was also built between the single scaffold-particles, however, bone formation within the whole defect did not differ significantly between the groups (Figure 3B).

**Figure 4:**
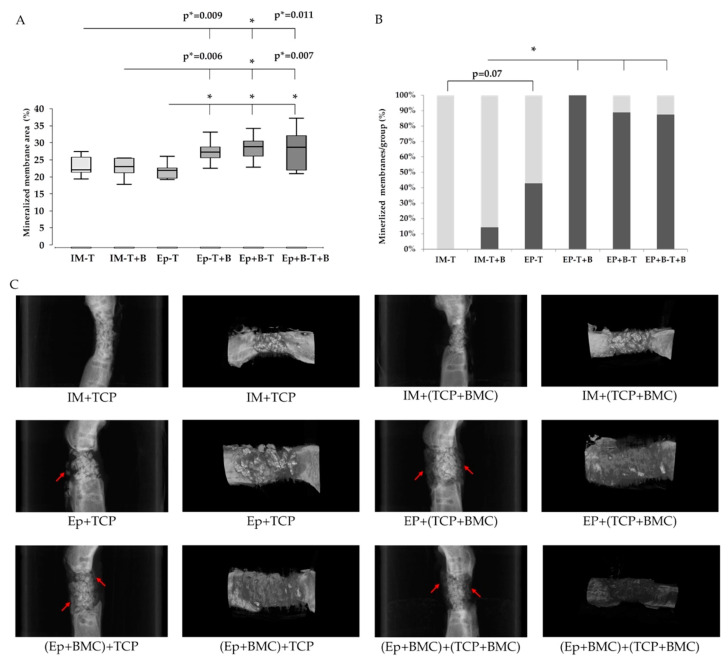
Confirmation of increased mineralization of the decellularized dermis in presence of BMC using µCT analysis (**A**). The percentage of samples with mineralized defect coating in each group is presented in (**B**). Representative µCT images are shown in (**C**). Left column: sagittal plane of the bone samples, arrows indicate mineralized dermis. Right column: surface reconstruction revealed deposition of mineralized material covering the scaffold granules in bone defects treated with decellularized dermis and BMC. * = *p* < 0.05. p* = uncorrected p-value, indicates a statistical trend.

The radiological analysis confirmed histological findings and the transformation of the implanted Epiflex^®^ into osseous tissue. In the presence of BMC, Epiflex^®^ showed significantly increased mineralization of the membrane area compared to groups without BMC. Furthermore, induced membranes (groups 1,2), independently of the presence of BMC, were distinctively less mineralized than Epiflex^®^ with BMC, reaching the level of significance in group 5 (Epiflex with BMC + β-TCP) (Figure 4A).

In groups with decellularized dermis and BMC, significantly higher percentage of samples showed mineralized defect coating; in group 4 (Epiflex with β-TCP + BMC), this was even up to 100% of the samples. However, in group 1 (Masquelet without BMC), no sample showed mineralized coating (Figure 4B).

Reconstructing the surface of the defects in µCT, groups with decellularized dermis and BMC showed mineralized material covering the scaffold granules within the defect zone (Figure 4C).

### 3.4. Callus Maturation

**Figure 5:**
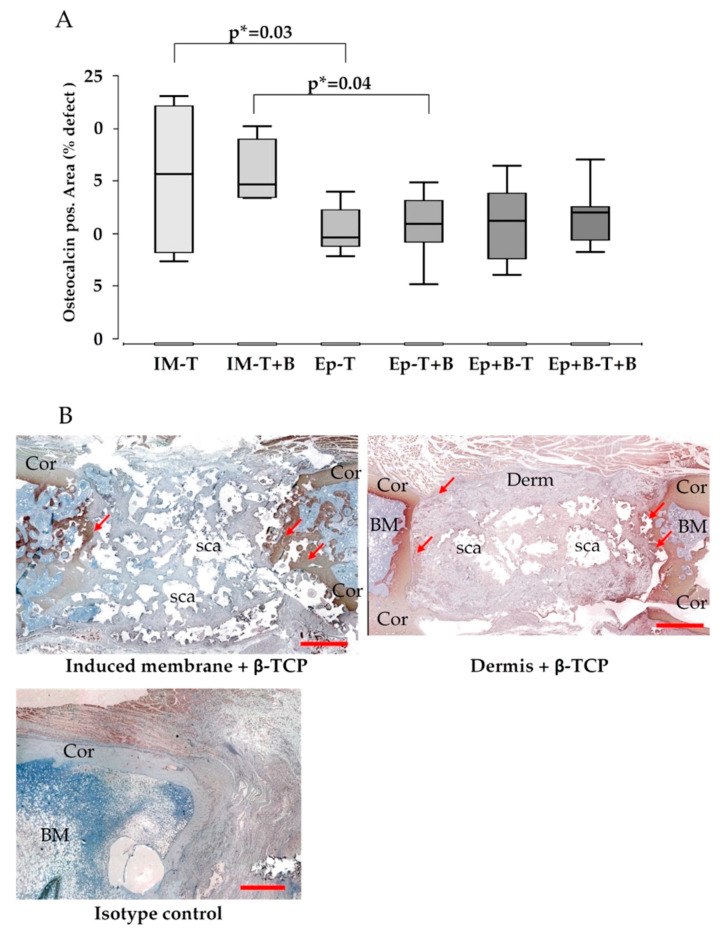
Data of histomorphometric evaluation of osteocalcin positive areas within the defect (**A**). Osteocalcin expression was increased in induced membrane-groups. Representative osteocalcin-stainings are shown in (**B**). Upper row left induced membrane, right decellularized dermis. Isotype control in the lower row. p* = uncorrected p-value, indicates a statistical trend. Cor = corticalis, Derm = decellularized dermis, IM = induced membrane, sca = scaffold. BM = bone marrow. Size bar upper row 2 mm, size bar isotype control 100 µm.

The maturation of the newly formed bone was assessed by histomorphometric evaluation of osteocalcin stained slides, and presented as osteocalcin positive percentage of the defect area (Figure 5A). Osteocalcin expression was increased in induced membrane-groups (groups 1,2), compared to Epiflex^®^-groups (group 3–6). However, a level of significance was not reached (Figure 5A,B).

### 3.5. Vascularization

**Figure 6:**
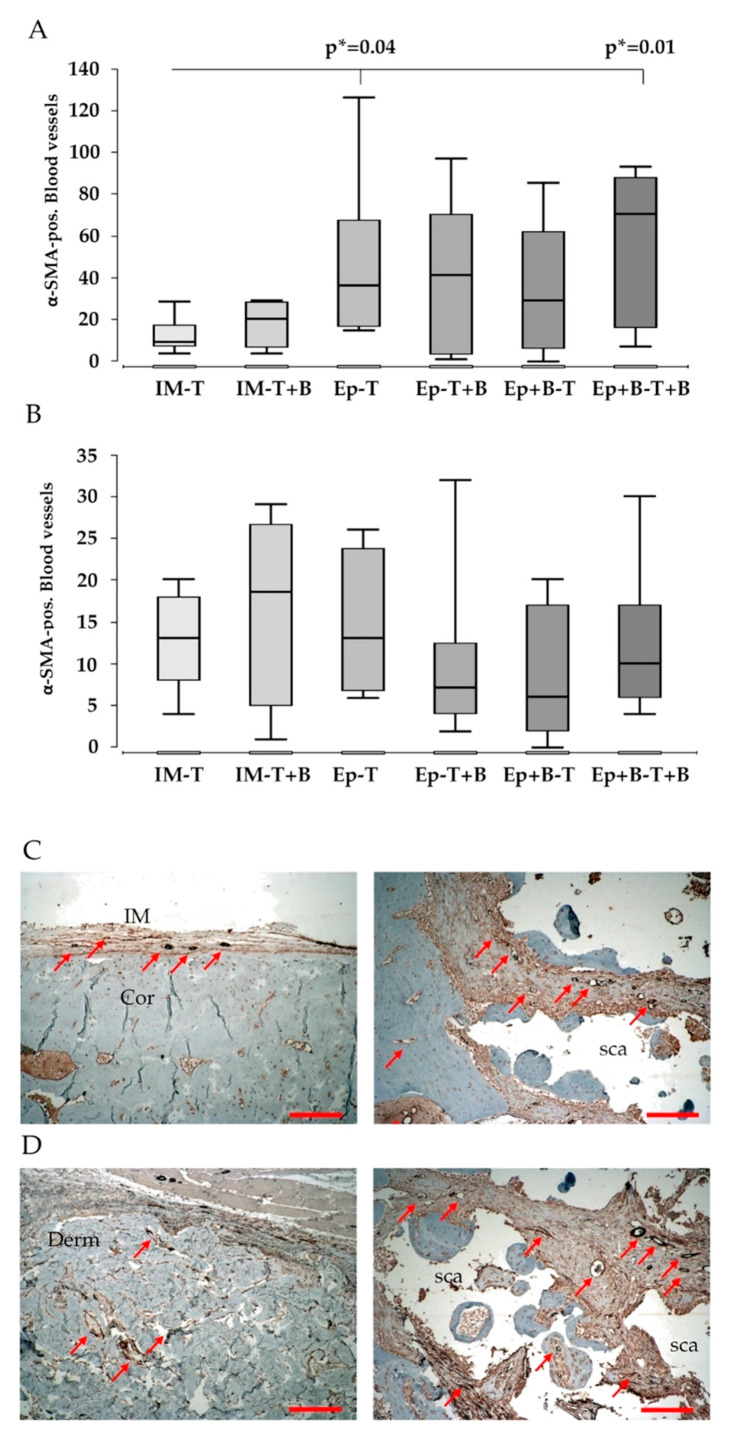
Evaluation of vascularization based on immunostaining of established blood vessel, using antibodies directed against α-SMA within the whole defect (**A**) and the induced membrane and decellularized dermis (**B**). Representative images of bone defects treated with an induced membrane (**C**) or decellularized dermis (**D**). Left images show the area of the induced membrane, respectively, of dermis, right images depict central defect areas. Notably, α-SMA-positive blood vessel in acellular dermis were predominantly seen in peripheral regions of the dermis in close vicinity to muscle, whereas α-SMA-positive vessels were generally not detectable in central portions of the decellularized dermis independently from the presence/absence of BMC within the dermis. In some cases, α-SMA-positive vessels, probably originating from muscle, were penetrating the acellular dermis. Moreover, α-SMA-positive vessels in the induced membrane were oftentimes more pronounced (established) compared to the decellularized dermis. p* = uncorrected p-value, indicates a statistical trend. Red arrows indicate α-SMA-positive blood vessels, Cor = corticalis, Derm = decellularized dermis, IM = induced membrane, sca = scaffold. Size bar indicates a distance of 200 µm.

No significant difference of vascularization was found between the membranes, respectively, in the dermis of the single groups. However, within the defect zone, groups with a decellularized dermis showed increased α-SMA-positive blood vessels compared to the Masquelet groups. The addition of BMC did not lead to an additional effect; defect zone of group 6 (Epiflex + BMC, β-TCP + BMC) contained most α-SMA-positive vessels, without reaching the level of significance (Figure 6A,B).

Generally, vessels showed no consistent distribution, but were concentrated in peripheral regions of the decellularized dermis, in close vicinity to the muscle. In central portions of the dermis no α-SMA-positive vessels were detectable, independently from the presence of BMC within the dermis. In some samples, α-SMA-positive vessels were penetrating into the dermis, originating from muscle tissue. In induced membranes, α-SMA-positive vessels were oftentimes more established compared to the dermis (Figure 6C).

## 4. Discussion

We aimed to investigate whether the induced membrane for the treatment of a critical size bone defect, as described by Masquelet et al. [4], can be replaced by an artificial membrane. We used, therefore, a decellularized human dermis (Epiflex^®^), repopulated with bone marrow derived mononuclear cells (BMC), and filled with either β-TCP or β-TCP loaded with BMC. A comparably good bone healing was observed with the one-stage technique, and the addition of BMC led to an improved mineralization. However, mechanically stable bone healing was not achieved within an eight weeks’ healing time. To the best of our knowledge, a decellularized skin graft as surrogate for the induced membrane, in combination with a scaffold, loaded with progenitor cells, has not been used before.

Epiflex^®^ is, analogously to the induced membrane, highly flexible, tear-proof and contains high amounts of elastic and structural fibers [29,30]. Epiflex^®^ is approved for multiple different indications, such as chronic skin wounds and burns [14,15], hernia surgery [16,17] or breast reconstruction [31]. Due to its intact native collagen structure, it is suitable as a matrix for angiogenesis, tissue regeneration and cell infiltration. It has been demonstrated that decellularized dermis can be easily vascularized by the engraftment of myeloid human endothelial progenitor cells (EPC), which develop from immature monocytes and hematopoietic stem cells [13]. The EPC populated dermis was shown to improve wound healing [18].

The function of the induced membrane is explained by two complementary hypotheses. On the one hand, it has a high osteogenic activity itself, due to the pre-established vascular network supporting vascularization of the implanted bone graft and the secretion of factors such as VEGF, BMP-2, and TGF-β, promoting bone regeneration. On the other hand, the membrane acts as a barrier to prevent surrounding soft tissue from growing into the defect and resorption of the implanted graft [6,32,33]. The osteogenic activity peaks between two and four weeks and mostly subsides after six weeks in rats, whereas in humans, a constant pro-osteogenic potential of the induced membrane could be seen over a period of up to 15 months [34]. However, the influence of vascularization of the induced membrane on bone regeneration seems to decline with increasing healing time [6,7,35]. The membrane then becomes a more capsule-like barrier surrounding the defect, acting passively as a bioreactor concentrating regenerative cells in the defect zone [6].

Epiflex^®^, by contrast, has no inert osteogenic activity, but, rather, serves as a matrix for cells to grow. However, in pretests, we were able to show that the dermis is able to absorb significant blood volume in the hydrated state and therefore a certain biologization of the membrane takes place during implantation [36].

Variations of Maquelet’s procedure are described numerously and approaches with an artificial dermis have been previously undertaken, for example, Liu et al. combined a PMMA spacer with an artificial dermis to support formation of Masquelet’s membrane [37]. The dermis disappeared completely after four weeks, and augmented membranes showed increased vascularization and membrane structure [37]. However, the principles of a two-stage procedure have remained untouched. Attempts to create a single-stage procedure are also described [38,39,40]. Tarchala et al. described a one-stage procedure and compared a non-biological membrane (polytetrafluoroethylene, PTFE) with the induced membrane. Finding comparable bone formation in both groups, they concluded—in line with Masquelet—that the membrane mainly works as a physical barrier [41,42]. However, the PTFE-membrane is not bioabsorbable and, thus, not feasible for clinical use.

Considering that the induced membrane’s quality of a physical barrier is most important for the success of Masquelet’s technique, the decellularized dermis provides this function right from the moment of implantation.

It is well-proven and already transferred to a clinical phase II-trial (EudraCT-Nr.:2015-001820-51), that cell-based therapy with BMC supports bone healing in our rat femoral bone defect model, significantly in combination with β-TCP as a bone void filler [10,26]. Those BMC are concentrated within the defect by the dermis surrounding it, preserving the cells from resorption.

Loading Epiflex^®^ and/or β-TCP with BMC did not significantly increase the vascularization of the dermis itself, as measured by the expression of α-SMA, in comparison to the induced membrane. However, the number of α-SMA-positive vessels within the defect zone was significantly increased in Epiflex^®^-groups, trending to be highest in groups with BMC-loaded β-TCP. Furthermore, the addition of BMC led to a significantly increased mineralization of the membrane in Epiflex^®^-groups compared to Masquelet-groups or cell-free Epiflex^®^-group (Figure 4A,B). This observation supports the aspect of the dermis working as a bioreactor, boosting the osteogenic effect of BMC. Histologically, the dermis itself showed a distinctly increased ossification when compared to the induced membrane, notably even more enhanced in groups with BMC (Figure 3). However, bone maturation, as measured by osteocalcin expression, was highest in Masquelet-groups, independently from BMC-addition.

Biomechanically, the newly formed bone was not stable enough for significant load bearing when harvested after a healing period of eight weeks, and only bending stiffness could be measured. Masquelet-groups showed significantly increased biomechanical properties compared to Epiflex^®^-groups (Figure 2A,B). In earlier work, our group found comparatively good biomechanical properties in a critical size defect-model of a rat femur treated with the induced membrane and β-TCP, with stem cells as bone void filler [7].

An explanation for the generally low biomechanical properties might be found in the interface between fracture site and Epiflex^®^. The decellularized dermis was fitted into the defect, placed under the plate and attached to it with a suture. In this way, the dermis was safely fixed around the defect, but no primary connection to the bone was achieved. The induced membrane, on the other hand, clinically and histologically seems to have a strong connection to the bone. However, in some of the samples, Epiflex^®^ showed an osseous connection to the fracture site (Figure 3C). In a further project, our group evaluated the decellularized dermis in a 5 mm femoral defect, with a slight overlap of 1 mm over the fracture site on both sides. A biomechanical analysis showed up to 63% of the resistance of the healthy contralateral femur [36]. In past studies, BMC have shown increased effect in smaller defects compared to larger defects [7,10]. However, apart from the smaller extent of the defect and the effectiveness of BMC, the improved interface between fracture site and dermis by a slight overlap must be considered as a reason for increased biomechanical results. Furthermore, a longer healing period could have resulted in more stable fracture healing. As also seen in earlier studies, bone formation in defects treated with the induced membrane mainly takes place from inside the defect by building osseous bridges between the single granules of the scaffold (Figure 3 and Figure 4) [7,43]. In contrast to this, defects treated with Epiflex^®^ not only showed bone formation between the scaffold particles, but mainly from the outside by mineralizing the membrane and, thus, forming a callus-like bone (Figure 3 and Figure 4).

## 5. Conclusions

A decellularized human dermis (Epiflex^®^) was used to replace Masquelet’s induced membrane and, therefore, eliminate the first surgical step, using a 10 mm femoral defect rat model. Defects treated with Epiflex^®^ showed increased mineralization and bone formation, predominantly in the transplanted dermis surrounding the defect, especially when BMC were added. Additionally, the vascularization of the defect could be enhanced by the addition of BMC. However, further studies are needed to establish the necessary conditions for this new bone healing technique, such as healing time.

The most important result of this study is that groups treated with Epiflex^®^ showed comparable and partially even significantly increased results, in comparison to conventional induced membrane groups. This leads to the conclusion that it might be feasible to eliminate the first step of Masquelet’s induced membrane technique in non-infected bones, by using a decellularized dermis in combination with regenerative cells (BMC).

If this model could be transferred successfully to a human model, this technique could have a great impact on clinical practice, resulting in a dramatic reduction of a patient’s burden, if—based on these results—the first surgical step and the time period to induce the membrane can be spared.

## Figures and Tables

**Table 1:** Experimental design and group set-up. IM induced membrane, BMC bone marrow mononuclear cells.

Group	Treatment	Number of Animals µCT/Biomechanics	Number of Animals for Histology	Donor-Animals for Syngenic BMC
1	IM + β-TCP	6	10	-
2	IM + (β-TCP + BMC)	6	10	4
3	Ep + β-TCP	6	10	-
4	Ep + (β-TCP + BMC)	6	10	4
5	(Ep + BMC) + β-TCP	6	10	4
6	(Ep + BMC) + (β-TCP + BMC)	6	10	4

## References

[1] Schemitsch E.H. (2017). Size Matters: Defining Critical in Bone Defect Size!. J. Orthop. Trauma..

[2] Keating J.F., Simpson A.H.R.W., Robinson C.M. (2005). The management of fractures with bone loss. J. Bone Jt. Surg. Br..

[3] Hannouche D., Petite H., Sedel L. (2001). Current trends in the enhancement of fracture healing. J. Bone Jt. Surg. Br..

[4] Masquelet A.C., Fitoussi F., Begue T., Muller G.P. (2000). Reconstruction of the long bones by the induced membrane and spongy autograft. Ann. Chir. Plast. Esthétique.

[5] Keramaris N.C., Kaptanis S., Moss H.L., Loppini M., Pneumaticos S., Maffulli N. (2012). Endothelial progenitor cells (EPCs) and mesenchymal stem cells (MSCs) in bone healing. Curr. Stem Cell Res. Ther..

[6] Henrich D., Seebach C., Nau C., Basan S., Relja B., Wilhelm K., Schaible A., Frank J., Barker J., Marzi I. (2016). Establishment and characterization of the Masquelet induced membrane technique in a rat femur critical-sized defect model. J. Tissue Eng. Regen. Med..

[7] Nau C., Simon S., Schaible A., Seebach C., Schröder K., Marzi I., Henrich D. (2018). Influence of the induced membrane filled with syngeneic bone and regenerative cells on bone healing in a critical size defect model of the rat’s femur. Injury.

[8] Janko M., Pöllinger S., Schaible A., Bellen M., Schröder K., Heilani M., Fremdling C., Marzi I., Nau C., Henrich D. (2020). Determination of the effective dose of bone marrow mononuclear cell therapy for bone healing in vivo. Eur. J. Trauma Emerg. Surg..

[9] Jeon O., Song S.J., Bhang S.H., Choi C.-Y., Kim M.J., Kim B.S. (2007). Additive effect of endothelial progenitor cell mobilization and bone marrow mononuclear cell transplantation on angiogenesis in mouse ischemic limbs. J. Biomed. Sci..

[10] Seebach C., Henrich D., Schaible A., Relja B., Jugold M., Bönig H., Marzi I. (2015). Cell-based therapy by implanted human bone marrow-derived mononuclear cells improved bone healing of large bone defects in rats. Tissue Eng. Part A.

[11] Nau C., Henrich D., Seebach C., Schröder K., Fitzsimmons S.J., Hankel S., Barker J.H., Marzi I., Frank J. (2016). Treatment of Large Bone Defects with a Vascularized Periosteal Flap in Combination with Biodegradable Scaffold Seeded with Bone Marrow-Derived Mononuclear Cells: An Experimental Study in Rats. Tissue Eng. Part A.

[12] Henrich D., Verboket R., Schaible A., Kontradowitz K., Oppermann E., Brune J.C., Nau C., Meier S., Bönig H., Marzi I. (2015). Characterization of bone marrow mononuclear cells on biomaterials for bone tissue engineering in vitro. BioMed Res. Int..

[13] Vitacolonna M., Belharazem D., Hohenberger P., Roessner E.D. (2017). In-vivo quantification of the revascularization of a human acellular dermis seeded with EPCs and MSCs in co-culture with fibroblasts and pericytes in the dorsal chamber model in pre-irradiated tissue. Cell Tissue Bank..

[14] Feng X., Tan J., Pan Y., Wu Q., Ruan S., Shen R., Chen X., Du Y. (2006). Control of hypertrophic scar from inception by using xenogenic (porcine) acellular dermal matrix (ADM) to cover deep second degree burn. Burns.

[15] Sín P., Brychta P. (2006). Cutometrical measurement confirms the efficacy of the composite skin grafting using allogeneic acellular dermis in burns. Acta Chir. Plast..

[16] Espinosa-de-los-Monteros A., de la Torre J.I., Marrero I., Andrades P., Davis M.R., Vásconez L.O. (2007). Utilization of human cadaveric acellular dermis for abdominal hernia reconstruction. Ann. Plast. Surg..

[17] Baillie D.R., Stawicki S.P., Eustance N., Warsaw D., Desai D. (2007). Use of human and porcine dermal-derived bioprostheses in complex abdominal wall reconstructions: A literature review and case report. Ostomy Wound Manag..

[18] Hendrickx B., Vranckx J.J., Luttun A. (2011). Cell-based vascularization strategies for skin tissue engineering. Tissue Eng. Part B Rev..

[19] Janko M., Dietz K., Rachor J., Sahm J., Schröder K., Schaible A., Nau C., Seebach C., Marzi I., Henrich D. (2019). Improvement of Bone Healing by Neutralization of microRNA-335-5p, but not by Neutralization of microRNA-92A in Bone Marrow Mononuclear Cells Transplanted into a Large Femur Defect of the Rat. Tissue Eng. Part A.

[20] Nau C., Seebach C., Trumm A., Schaible A., Kontradowitz K., Meier S., Buechner H., Marzi I., Henrich D. (2016). Alteration of Masquelet“s induced membrane characteristics by different kinds of antibiotic enriched bone cement in a critical size defect model in the rat’s femur. Injury.

[21] Verboket R., Leiblein M., Seebach C., Nau C., Janko M., Bellen M., Bonig H., Henrich D., Marzi I. (2018). Autologous cell-based therapy for treatment of large bone defects: From bench to bedside. Eur. J. Trauma Emerg. Surg..

[22] Seebach C., Henrich D., Meier S., Nau C., Bönig H., Marzi I. (2016). Safety and feasibility of cell-based therapy of autologous bone marrow-derived mononuclear cells in plate-stabilized proximal humeral fractures in humans. J. Transl. Med..

[23] Vitacolonna M., Belharazem D., Hohenberger P., Roessner E.D. (2015). Effect of dynamic seeding methods on the distribution of fibroblasts within human acellular dermis. Cell Tissue Bank..

[24] Seebach C., Henrich D., Kähling C., Wilhelm K., Tami A.E., Alini M., Marzi I. (2010). Endothelial progenitor cells and mesenchymal stem cells seeded onto beta-TCP granules enhance early vascularization and bone healing in a critical-sized bone defect in rats. Tissue Eng. Part A.

[25] Henrich D., Seebach C., Kähling C., Scherzed A., Wilhelm K., Tewksbury R., Powerski M., Marzi I. (2009). Simultaneous cultivation of human endothelial-like differentiated precursor cells and human marrow stromal cells on beta-tricalcium phosphate. Tissue Eng. Part C Methods.

[26] Janko M., Sahm J., Schaible A., Brune J.C., Bellen M., Schröder K., Seebach C., Marzi I., Henrich D. (2017). Comparison of three different types of scaffolds preseeded with human bone marrow mononuclear cells on the bone healing in a femoral critical size defect model of the athymic rat. J. Tissue Eng. Regen. Med..

[27] Garvey W., Fathi A., Bigelow F., Carpenter B., Jimenez C. (1986). Improved Movat pentachrome stain. Stain. Technol..

[28] Henrich D., Seebach C., Verboket R., Schaible A., Marzi I., Bonig H. (2018). The osteo-inductive activity of bone-marrow-derived mononuclear cells resides within the CD14+ population and is independent of the CD34+ population. Eur. Cells Mater..

[29] Anderson J.M., Rodriguez A., Chang D.T. (2008). Foreign body reaction to biomaterials. Semin. Immunol..

[30] Sorrell J.M., Caplan A.I. (2009). Fibroblasts-a diverse population at the center of it all. Int. Rev. Cell Mol. Biol..

[31] Holton L.H., Haerian H., Silverman R.P., Chung T., Elisseeff J.H., Goldberg N.H., Slezak S. (2005). Improving long-term projection in nipple reconstruction using human acellular dermal matrix: An animal model. Ann. Plast. Surg..

[32] Gaio N., Martino A., Toth Z., Watson J.T., Nicolaou D., McBride-Gagyi S. (2018). Masquelet technique: The effect of altering implant material and topography on membrane matrix composition, mechanical and barrier properties in a rat defect model. J. Biomech..

[33] Wang W., Zuo R., Long H., Wang Y., Zhang Y., Sun C., Luo G., Zhang Y., Li C., Zhou Y. (2020). Advances in the Masquelet technique: Myeloid-derived suppressor cells promote angiogenesis in PMMA-induced membranes. Acta Biomater..

[34] Gindraux F., Loisel F., Bourgeois M., Oudina K., Melin M., de Billy B., Sergent P., Leclerc G., Petite H., Auber F. (2020). Induced membrane maintains its osteogenic properties even when the second stage of Masquelet’s technique is performed later. Eur. J. Trauma Emerg. Surg..

[35] Aho O.M., Lehenkari P., Ristiniemi J., Lehtonen S., Risteli J., Leskelä H.V. (2013). The mechanism of action of induced membranes in bone repair. J. Bone Jt. Surg. Am..

[36] Verboket R.D., Leiblein M., Janko M., Schaible A., Brune J.C., Schröder K., Heilani M., Fremdling C., Busche Y., Irrle T. (2020). From two stages to one: Acceleration of the induced membrane (Masquelet) technique using human acellular dermis for the treatment of non-infectious large bone defects. Eur. J. Trauma Emerg. Surg..

[37] Liu K., Wang Y., Sun Y., Qi X., Tian L., Zhao Y., Xu Y., Liu X. (2019). [Masquelet technique combined with artificial dermis for the treatment of bone and soft tissue defects in rabbits]. Zhongguo Xiu Fu Chong Jian Wai Ke Za Zhi.

[38] DeBaun M.R., Stahl A.M., Daoud A.I., Pan C.C., Bishop J.A., Gardner M.J., Yang Y.P. (2019). Preclinical induced membrane model to evaluate synthetic implants for healing critical bone defects without autograft. J. Orthop. Res..

[39] Meinig R.P., Rahn B., Perren S.M., Gogolewski S. (1996). Bone regeneration with resorbable polymeric membranes: Treatment of diaphyseal bone defects in the rabbit radius with poly(L-lactide) membrane. A pilot study. J. Orthop. Trauma.

[40] Gindraux F., Rondot T., de Billy B., Zwetyenga N., Fricain J.C., Pagnon A., Obert L. (2017). Similarities between induced membrane and amniotic membrane: Novelty for bone repair. Placenta.

[41] Tarchala M., Engel V., Barralet J., Harvey E.J. (2018). A pilot study: Alternative biomaterials in critical sized bone defect treatment. Injury.

[42] Masquelet A.C., Begue T. (2010). The concept of induced membrane for reconstruction of long bone defects. Orthop. Clin. N. Am..

[43] Leiblein M., Koch E., Winkenbach A., Schaible A., Nau C., Büchner H., Schröder K., Marzi I., Henrich D. (2020). Size matters: Effect of granule size of the bone graft substitute (Herafill^®^) on bone healing using Masquelet’s induced membrane in a critical size defect model in the rat’s femur. J. Biomed. Mater. Res. Part B Appl. Biomater..

